# Obinutuzumab maintenance versus observation for patients with newly diagnosed high tumor burden follicular lymphoma who achieved complete metabolic response after obinutuzumab plus bendamustine induction therapy: a multicenter, randomized, phase III study (JCOG2008, MAIN study)

**DOI:** 10.1093/jjco/hyaf038

**Published:** 2025-02-25

**Authors:** Tsutomu Kobayashi, Kenichi Ishizawa, Ryunosuke Machida, Ryo Sadachi, Keita Sasaki, Haryoon Kim, Keisuke Kataoka, Wataru Munakata, Noriko Fukuhara, Hirokazu Nagai, Lymphoma Study Group of the Japan Clinical Oncology Group

**Affiliations:** Department of Hematology, Japanese Red Cross Kyoto Daiichi Hospital, 15-749 Honmachi, Higashiyama-ku, Kyoto 605-0981, Japan; Department of Nursing, Faculty of Health Sciences, Tohoku Fukushi University, 1-8-1 Kunimi Aoba-ku Sendai-shi Miyagi, 981-8522, Japan; Japan Clinical Oncology Group Data Center/Operations Office, National Cancer Center Hospital, 5-1-1 Tsukiji, Chuo-ku, Tokyo 104-0045, Japan; Japan Clinical Oncology Group Data Center/Operations Office, National Cancer Center Hospital, 5-1-1 Tsukiji, Chuo-ku, Tokyo 104-0045, Japan; Japan Clinical Oncology Group Data Center/Operations Office, National Cancer Center Hospital, 5-1-1 Tsukiji, Chuo-ku, Tokyo 104-0045, Japan; Division of Hematology, Department of Medicine, Keio University School of Medicine, 35 Shinanomachi, Shinjuku-ku, Tokyo 160-8582, Japan; Division of Molecular Oncology, National Cancer Center Research Institute, 5-1-1 Tsukiji, Chuo-ku, Tokyo 104-0045, Japan; Division of Hematology, Department of Medicine, Keio University School of Medicine, 35 Shinanomachi, Shinjuku-ku, Tokyo 160-8582, Japan; Division of Molecular Oncology, National Cancer Center Research Institute, 5-1-1 Tsukiji, Chuo-ku, Tokyo 104-0045, Japan; Department of Hematology, National Cancer Center Hospital, 5-1-1 Tsukiji, Chuo-ku, Tokyo 104-0045, Japan; Department of Hematology, Tohoku University Graduate School, 2-1-1 Katahira, Aoba-ku, Sendai, Miyagi 980-8577, Japan; Department of Hematology and Oncology, National Hospital Organization Nagoya Medical Center, 1-1 Sannomaru 4-chome, Naka-ku, Nagoya 460-0001, Japan

**Keywords:** follicular lymphoma, maintenance therapy, obinutuzumab, bendamustine, complete metabolic response

## Abstract

Maintenance therapy with monoclonal anti-CD20 antibody is the standard approach in patients with follicular lymphoma who initially treated and achieved response to immunochemotherapy. Maintenance therapy reduces the risk of lymphoma progression, but the risk of late or delayed fatal treatment-emergent adverse events is a clinically important issue. The aim of this randomized phase III study is to confirm the non-inferiority of observation compared to obinutuzumab maintenance therapy in patients with untreated high tumor burden follicular lymphoma who achieved complete metabolic response after obinutuzumab plus bendamustine induction therapy (JCOG2008, MAIN study). The first registration is performed before obinutuzumab plus bendamustine administration. Those who achieved complete metabolic response at the end of induction are included in the second registration and randomized to an obinutuzumab maintenance arm or observation only. This study has been registered in the Japan Registry for Clinical Trials as jRCT1031210379.

## Teaser

This study aims to determine whether observation is non-inferior to obinutuzumab maintenance therapy in patients with previously untreated follicular lymphoma with complete metabolic response after obinutuzumab plus bendamustine induction therapy.

## Introduction

Follicular lymphoma (FL) accounts for ~20% of cases of non-Hodgkin lymphoma as the second most common lymphoma and is one of the most representative indolent B-cell lymphomas [1]. Use of a monoclonal anti-CD20 antibody, rituximab, has markedly improved the treatment outcomes of patients with newly diagnosed FL [2], and rituximab combined with chemotherapy has been the standard treatment since early 2000. Obinutuzumab, a recently approved novel glycoengineered type-2 monoclonal anti-CD20 antibody, induces enhanced antibody-dependent cell cytotoxicity and direct cell death compared with rituximab [3]. A pivotal clinical study (GALLIUM) demonstrated that obinutuzumab-based immunochemotherapy and maintenance therapy had longer progression-free survival (PFS) than rituximab-based therapy [3-year PFS, 80.0 vs. 73.3%, hazard ratio (HR) 0.66, *P* = 0.001] [4]. In combination chemotherapy with rituximab or obinutuzumab, bendamustine tends to give a longer PFS than CHOP or CVP [5]; therefore, obinutuzumab and bendamustine (OB) therapy is the most commonly used regimen for patients with newly diagnosed high tumor burden FL in Japan.

Although obinutuzumab-based therapy is the most promising induction therapy for patients with previously untreated FL in terms of prolonged PFS, the risk of treatment-emergent adverse events is a clinically important problem. In fact, adverse events of Grade 3 or higher, as defined in the Common Terminology Criteria for Adverse Events (CTCAE), occur more frequently after obinutuzumab-based therapy than rituximab-based therapy; e.g. infectious events (20 vs. 16%), neutropenia (46 vs. 40%), and thrombocytopenia (6 vs. 3%) [4]. Furthermore, fatal adverse events are most frequent after bendamustine combined with anti-CD20 monoclonal antibody therapy, and especially with obinutuzumab plus bendamustine therapy, occurring at a rate of 6% of cases [5].

Maintenance therapy using anti-CD20 monoclonal antibody gives prolonged PFS compared to observation only in patients with FL, as confirmed in the PRIMA study [6]. This pivotal study demonstrated that rituximab maintenance therapy after R-CHOP/CVP significantly prolonged PFS in long-term follow-up (median PFS, 10.5 vs. 4.1 years, HR 0.61, *P* < 0.001), which resulted in adoption of rituximab maintenance therapy as the standard strategy [7]. Regarding bendamustine-based immunochemotherapy, there are several reports showing the effectiveness of maintenance therapy in patients with FL [8,9]. In addition, Luminari et al. showed the importance of rituximab maintenance therapy even in patients with newly diagnosed FL who achieved complete metabolic response (CMR) and had undetectable minimal residual disease after the rituximab combined induction chemotherapy [10].

Despite these promising results, there are several problems with maintenance therapy. First, this therapy has no overall survival (OS) benefit [7,9,10]. Second, maintenance therapy has been reported to increase the risk of infection by reducing lymphocytes and delaying recovery [11,12]. Of note, no prospective study to date has shown the benefits of obinutuzumab maintenance. In addition, the significance of maintenance therapy may differ depending on the therapeutic regimen or the response at the end of induction therapy [13]. In the report by Hill et al., maintenance therapy with rituximab did not provide a survival benefit in patients with FL with CMR after induction therapy compared to observation only. Thus, it is unclear if maintenance therapy is necessary in all cases of FL after induction therapy.

Based on the above evidence, we hypothesized that the PFS benefit of maintenance therapy is largely confined to insufficient responders who remain PET-positive after induction therapy, especially if this is bendamustine-based, and is of limited benefit for patients who achieve CMR. Therefore, it may be possible to omit maintenance therapy for patients with newly diagnosed FL with CMR after OB therapy to reduce the recurrence risk.

**Figure 1:**
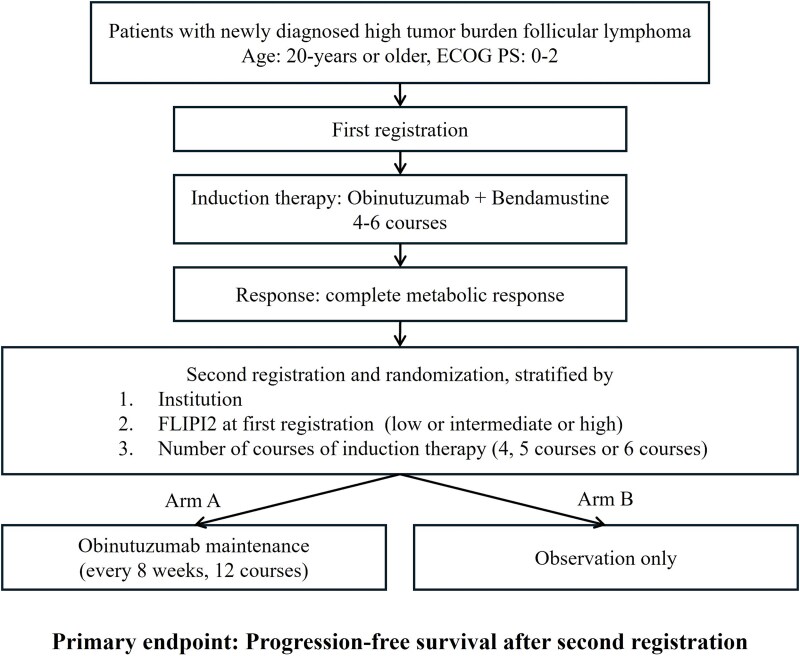
**Study schema of JCOG2008.** ECOG PS, European Clinical Oncology Group Performance Status; FLIPI2, follicular lymphoma international prognostic index 2.

JCOG2008 was planned to examine this hypothesis. The schema of JCOG2008 is shown in Fig. 1. The JCOG Protocol Review Committee approved the JCOG2008 protocol in July 2021, and the Certified Review Board of the National Cancer Center approved the study in August 2021. Patient accrual was started from October 2021. The trial has been registered in the Japan Registry for Clinical Trials as jRCT1031201379 (https://jrct.niph.go.jp/).

## Protocol digest of the JCOG2008

### Objectives

The aim of the trial is to confirm the non-inferiority of observation to obinutuzumab maintenance in patients with newly diagnosed high tumor burden FL who achieved CMR after OB induction therapy.

### Study setting

A multi-institutional, two-arm, open-label, randomized phase III study.

### Endpoints

The primary endpoint is PFS after the second registration. PFS is defined as the time from registration to the earliest date of progression or death from any cause and will be censored at the latest day when the patient is confirmed to be alive without any evidence of progression. The secondary endpoints are OS after the first registration, OS after the second registration, time to next treatment (TTNT) from the first registration, TTNT from the second registration, PFS after the first registration, proportion of progression of disease within 24 months after the first registration, overall response rate, complete response rate, and CMR rate of induction therapy, adverse events, severe adverse events, and proportion of occurrence of histological/clinical transformation. OS is defined as the time from the date of registration to death from any cause and will be censored at the last day the patient is confirmed to be alive.

### Eligibility criteria

#### Inclusion criteria

*First registration criteria.*

(1) FL (Grade 1, 2, or 3A) with a CD20-positive status.

(2) Ann Arbor Clinical Stage II, III, or IV diagnosed by CT and bone marrow examination.

(3) Any of the following (i)–(viii) in physical findings and CT within 56 days before registration:

(i) Largest nodal or extranodal mass with a diameter ≥ 7 cm.

(ii) More than three nodal sites with a diameter ≥ 3 cm.

(iii) Lymphoma cell count in peripheral blood >5000/mm^3^.

(iv) Presence of B symptoms.

(v) Spleen size ≥16 cm on CT.

(vi) Massive serous effusions.

(vii) Critical organ compression.

(viii) Cytopenia due to lymphomatous bone marrow involvement, based on fulfilling one or more of the following three criteria: absolute neutrophil count <1000/mm^3^; hemoglobin concentration < 10 g/dL; absolute platelet count <100 000/mm^3^.

(4) Age ≥ 20 years old.

(5) European Clinical Oncology Group (ECOG) performance status (PS) of 0–2.

(6) Measurable lesions.

(7) No clinical central nervous system involvement.

(8) No prior chemotherapy, radiotherapy, interferon-alfa, or antibody therapy.

(9) Patients with sufficient organ function.

(10) Written informed consent.

*Second registration criteria.*

(1) First registration for this trial.

(2) Satisfying (i) or (ii) as follows: (i) six courses of OB therapy completed and CMR obtained on FDG-PET/CT; (ii) only four or five courses of OB therapy completed due to adverse events and CMR obtained on FDG-PET/CT.

(3) No non-hematological toxicity of Grade 2 or higher.

(4) ECOG PS of 0–1.

(5) Patients with sufficient organ function.

(6) Within 42–98 days from the last administration of OB therapy.

#### Exclusion criteria

(1) Synchronous double or multiple cancer or metachronous double or multiple cancer with a progression-free period <5 years.

(2) Infectious disease requiring systemic treatment.

(3) Fever >38.0°C (except when infection or drug fever can be ruled out)

(4) Female patient during pregnancy, within 28 days of postparturition, or during lactation, or a male patient expecting a partner's pregnancy.

(5) Severe psychological disorder.

(6) Receiving continuous systemic corticosteroids or immunosuppressant treatment.

(7) Uncontrolled diabetes mellitus.

(8) Uncontrolled hypertension.

(9) Unstable angina pectoris or history of myocardial infarction within 6 months.

(10) HBs-Ag positive or HCV-Ab positive.

(11) HBs-Ab and/or HBc-Ab positive with a HBV DNA level ≥ 20 IU/mL (1.3 logIU/mL)

(12) Positive for HIV antibody.

### Randomization

After confirming fulfillment of the second registration criteria, registration is performed using a web-based system at the JCOG Data Center. Patients are randomized to arm A (obinutuzumab maintenance) or arm B (observation only) with a minimization method balancing the arms for institution, FL international prognostic index 2 (FLIPI2) (low-risk vs. intermediate-risk vs. high-risk) [14] at the first registration, and the number of courses of induction therapy (4–5 vs. 6 courses).

### Treatment methods

Induction therapy: ***OB therapy (every 4 weeks).*** Course 1: obinutuzumab 1000 mg/body (Days 1, 8, 15), bendamustine 90 mg/m^2^ (Days 1, 2). Courses 2–6: obinutuzumab 1000 mg/body (Day 1), bendamustine 90 mg/m^2^ (Days 1, 2).

Maintenance therapy: ***Arm A: Obinutuzumab maintenance therapy.*** Obinutuzumab 1000 mg/body (Day 1), every 8 weeks, 12 courses in total. ***Arm B: Observation.*** Patients receive follow-up observation.

### Follow-up

All randomized patients will be followed-up for at least 5 years after the second registration is completed. Blood tests to evaluate safety of the patients are performed after every course during the induction phase, every course during the maintenance phase in arm A, and every 8 weeks during the first year from completion of protocol treatment and every 12 weeks during the next year in arm B. FDG-PET/CT to evaluate the response to induction therapy is performed within 42–70 days from the last administration of OB therapy. A bone marrow test and/or gastrointestinal endoscopy is also performed in patients with bone marrow and/or gastrointestinal involvement at the first registration. Enhanced CT to evaluate the response after maintenance therapy is performed every 24 weeks during the first 5 years from the second registration, and every 48 weeks thereafter. All adverse events will be evaluated using CTCAE ver. 5.0.

### Study design and statistical analyses

This trial is designed to confirm the non-inferiority of arm B to arm A in terms of PFS after the second registration. The primary analyses will be conducted 5 years after completion of the second registration. The HR for PFS after the second registration between arms and the confidence interval will be estimated using a Cox proportional hazard model stratified by FLIPI2 (low-risk vs. intermediate-risk vs. high-risk) at the first registration and the number of courses of induction therapy (4–5 vs. 6 courses). If the upper limit of the confidence interval after adjusting for multiplicity is lower than the non-inferiority margin of 1.46 in the HR, we will conclude that observation only is an option in patients with newly diagnosed high tumor burden FL who achieved CMR after obinutuzumab plus bendamustine induction therapy.

The 2-year PFS after the second registration is assumed to be 88% in both arms A and B and the non-inferiority margin is set at 5% (HR 1.46). According to the Schoenfeld and Richter method [15], the required sample size was calculated as 352 patients to observe 132 events with a one-sided alpha of 5%, a statistical power of 70%, an accrual period of 5 years, and a follow-up period of 5 years. The total sample size for the second registration was set at 360 patients, assuming some loss to follow-up. All statistical analyses will be conducted at the JCOG Data Center.

### Interim analyses and monitoring

We plan to conduct two interim analyses. The first will be used to determine whether continuing patient accrual is reasonable, after enrollment of 50% of the planned number of patients in the second registration. The second analysis will be performed to determine whether follow-up should be continued, at ~6 months after patient accrual is completed. Multiplicity adjustment will be performed using the Lan-DeMets method with an O’Brien and Fleming-type alpha spending function [16]. The JCOG Data and Safety Monitoring Committee will review the interim analysis reports independently from the study investigators and a study statistician to determine if the trial should be terminated early. The trial will be terminated for futility (i) when the estimated HR of PFS in arm B to that in arm A is greater than the non-inferiority margin (HR: 1.46) or (ii) when PFS in arm B is inferior to that in arm A (the decision will be made comprehensively, rather than based on a statistical test). The JCOG Data Center and Study Coordinator will conduct central monitoring and the JCOG Data Center will issue a monitoring report every 6 months to evaluate the study progress and improve data integrity and patient safety. For quality assurance, site visit audits will be performed by the JCOG Audit Committee (not on a study-specific basis, but for the study group).

## JCOG2008A1: exploratory biomarker study of JCOG2008

With the advent of bendamustine and obinutuzumab, the utility of conventional prognostic indices, such as FLIPI and FLIPI2, has become controversial. Recently, several molecular risk stratification methods have been reported, including m7-FLIPI, which integrates PS, FLIPI score, and the mutational status of seven genes [17], and LYSA23 [18]. However, the prognostic values of these methods have not been widely examined and are considered to depend on therapeutic regimens. Therefore, we planned and are conducting an ancillary study (JCOG2008A1) using next-generation sequencing analysis and nCounter digital gene expression analysis to investigate the relevance of conventional clinicogenomic prognostic models in FL patients treated with OB therapy, and to explore new genetic abnormalities or gene expression levels that may be useful for prognostic prediction in high tumor burden FL.

### DNA and RNA extraction

Formalin-fixed paraffin-embedded (FFPE) samples from patients with high tumor burden FL will be collected prior to OB induction therapy. Subsequently, genomic DNA and RNA of tumor tissues will be extracted at the central laboratory for further genetic analyses.

### Targeted capture sequencing

Using the genomic DNA extracted from FFPE samples, targeted capture sequencing of 322 genes will be performed. These 322 genes were chosen because they have been reported to be clinically important in malignant lymphomas, including FL. Associations of genetic alterations detected by targeted capture sequencing with (i) baseline clinicopathological characteristics and (ii) treatment outcomes will be investigated.

### nCounter digital gene expression analysis

Using the RNA extracted from FFPE samples, nCounter digital gene expression analysis of 620 genes will be performed. These 620 genes comprise 579 immune-related genes, 23 genes included in LYSA23, and 18 internal control genes. Associations of gene expression data obtained from nCounter digital gene expression analysis with (i) baseline clinicopathological characteristics and (ii) treatment outcomes will be investigated.

## Participating institutions (from north to south)

Sapporo Hokuyu Hospital.

Iwate Medical University.

Tohoku University Hospital.

Akita University Hospital.

Yamagata University Hospital.

Tsukuba University Hospital.

Gunma University Hospital.

Saitama Medical University International Medical Center.

Saitama Medical Center.

National Cancer Center Hospital East.

Chiba Cancer Center.

National Cancer Center Hospital.

Kyorin University Faculty of Medicine.

Tokyo Metropolitan Cancer and Infectious Disease Center Komagome Hospital.

Keio University Hospital.

Jikei University Hospital.

Jikei University Daisan Hospital.

Cancer Institute Hospital, Japanese Foundation for Cancer Research.

Tokai University School of Medicine.

Kanagawa Cancer Center.

University of Fukui Hospital.

Gifu University School of Medicine.

Aichi Cancer Center Hospital.

National Hospital Organization Nagoya Medical Center.

Nagoya University School of Medicine.

Fujita Health University.

Nagoya City University Hospital.

Japanese Red Cross Aichi Medical Center Nagoya Daini Hospital.

Aichi Medical University.

Toyota Kosei Hospital.

Mie University School of Medicine.

Kyoto Prefectural University of Medicine.

Japanese Red Cross Kyoto Daiichi Hospital.

Osaka University Hospital.

Kindai University Hospital.

Osaka International Cancer Institute.

Hyogo Medical University.

Hyogo Cancer Center.

Wakayama Medical University.

Shimane University of Medicine.

National Hospital Organization Shikoku Cancer Center.

National Kyushu Cancer Center.

Fukuoka University School of Medicine.

University of Occupational and Environmental Health, Faculty of Medicine.

Saga University School of Medicine.

National Hospital Organization Nagasaki Medical Center.

Sasebo City General Hospital.

Nagasaki University Hospital.

Kumamoto University Hospital.

National Hospital Organization Kumamoto Medical Center.

Oita Prefectural Hospital.

Kagoshima University, Faculty of Medicine.

University of the Ryukyus Hospital.

## References

[1] Chihara D, Ito H, Matsuda T. et al. Differences in incidence and trends of haematological malignancies in Japan and the United States. Br J Haematol 2014;164:536–45. 10.1111/bjh.12659. 24245986 PMC3907701

[2] Hiddemann W, Kneba M, Dreyling M. et al. Frontline therapy with rituximab added to the combination of cyclophosphamide, doxorubicin, vincristine, andprednisone (CHOP) significantly improves the outcome for patients with advanced-stage follicular lymphoma compared with therapy with CHOP alone: results of a prospective randomized study of the German Low-Grade Lymphoma Study Group. Blood 2005;106:3725–32. 10.1182/blood-2005-01-0016. 16123223

[3] Tobinai K, Klein C, Oya N, Fingerle-Rowson G. A review of obinutuzumab (GA101), a novel type II anti-CD20 monoclonal antibody, for the treatment of patients with B-cell malignancies. Adv Ther 2017;34:324–56. 10.1007/s12325-016-0451-1. 28004361 PMC5331088

[4] Marcus R, Davies A, Ando K. et al. Obinutuzumab for the first-line treatment of follicular lymphoma. N Engl J Med 2017;377:1331–44. 10.1056/NEJMoa1614598. 28976863

[5] Hiddemann W, Barbui AM, Canales MA. et al. Immunochemotherapy with obinutuzumab or rituximab for previously untreated follicular lymphoma in the GALLIUM study: influence of chemotherapy on efficacy and safety. J Clin Oncol 2018;36:2395–404. 10.1200/JCO.2017.76.8960. 29856692

[6] Salles G, Seymour JF, Offner F. et al. Rituximab maintenance for 2 years in patients with high tumour burden follicular lymphoma responding to rituximab plus chemotherapy (PRIMA): a phase 3, randomised controlled trial. Lancet 2011;377:42–51. 10.1016/S0140-6736(10)62175-7. 21176949

[7] Bachy E, Seymour JF, Feugier P. et al. Sustained progression-free survival benefit of rituximab maintenance in patients with follicular lymphoma: long-term results of the PRIMA study. J Clin Oncol 2019;37:2815–24. 10.1200/JCO.19.01073. 31339826 PMC6823890

[8] Flinn IW, van der Jagt R, Kahl BS. et al. Randomized trial of bendamustinerituximab or R-CHOP/R-CVP in first-line treatment of indolent NHL or MCL: the BRIGHT study. Blood 2014;123:2944–52. 10.1182/blood-2013-11-531327. 24591201 PMC4260975

[9] Flinn IW, van der Jagt R, Kahl BS. et al. First-line treatment of patients with indolent non-Hodgkin lymphoma or mantle-cell lymphoma with bendamustine plus rituximab versus R-CHOP or R-CVP: results of the BRIGHT 5-year follow-up study. J Clin Oncol 2019;37:984–91. 10.1200/JCO.18.00605. 30811293 PMC6494265

[10] Luminari S, Manni M, Galimberti S. et al. Response-adapted postinduction strategy in patients with advanced-stage follicular lymphoma: the FOLL12 study. J Clin Oncol 2022;40:729–39. 10.1200/JCO.21.01234. 34709880

[11] Vidal L, Gafter-Gvili A, Salles G. et al. Rituximab maintenance for the treatment of patients with follicular lymphoma: an updated systematic review and meta-analysis of randomized trials. J Natl Cancer Inst 2011;103:1799–806. 10.1093/jnci/djr418. 22021664

[12] Yutaka T, Ito S, Ohigashi H. et al. Sustained CD4 and CD8 lymphopenia after rituximab maintenance therapy following bendamustine and rituximab combination therapy for lymphoma. Leuk Lymphoma 2015;56:3216–8. 10.3109/10428194.2015.1026818. 25760636

[13] Hill BT, Nastoupil L, Winter AM. et al. Maintenance rituximab or observation after frontline treatment with bendamustine-rituximab for follicular lymphoma. Br J Haematol 2019;184:524–35. 10.1111/bjh.15720. 30575016 PMC6486816

[14] Federico M, Bellei M, Marcheselli L. et al. Follicular lymphoma international prognostic index 2: a new prognostic index for follicular lymphoma developed by the international follicular lymphoma prognostic factor project. J Clin Oncol 2009;27:4555–62. 10.1200/JCO.2008.21.3991. 19652063

[15] Schoenfeld DA, Richter JR. Nomograms for calculating the number of patients needed for a clinical trial with survival as an endpoint. Biometrics 1982;38:163–70. 10.2307/2530299. 7082758

[16] Lan KKG, DeMets DL. Discrete sequential boundaries for clinical trials. Biometrika 1983;70:659–63. 10.2307/2336502.

[17] Pastore A, Jurinovic V, Kridel R. et al. Integration of gene mutations in risk prognostication for patients receiving first-line immunochemotherapy for follicular lymphoma: a retrospective analysis of a prospective clinical trial and validation in a population-based registry. Lancet Oncol 2015;2045:1–12. 10.1016/S1470-2045(15)00169-2. 26256760

[18] Huet S, Tesson B, Jais JP. et al. A gene-expression profiling score for prediction of outcome in patients with follicular lymphoma: a retrospective training and validation analysis in three international cohorts. Lancet Oncol 2018;19:549–61. 10.1016/S1470-2045(18)30102-5. 29475724 PMC5882539

